# Self-managed abortion as a humanitarian revolution: accounts of a telehealth pilot in the Middle East

**DOI:** 10.1186/s13031-024-00641-1

**Published:** 2025-02-11

**Authors:** Laureline Lasserre, Nelly Staderini, Maysa’a Hasan, Vanessa Rossi

**Affiliations:** Médecins Sans Frontières, Geneva, Switzerland

**Keywords:** Safe abortion care, Middle East, Self-management, Telehealth

## Abstract

**Background:**

Access to safe abortion care (SAC) should be improved in fragile and humanitarian settings, and the implementation of interventions in that regard are currently limited. This is especially true for self-managed abortion (SMA), although it holds the potential of revolutionizing the prevention of maternal death and suffering.

**Case presentation:**

The medical humanitarian organization Doctors Without Borders/Médecins Sans Frontières (MSF) piloted a self-managed abortion model of care in the Middle East. 22 women were remotely supported in managing their safe abortions with a counsellor over the phone, using misoprostol doses that they took at home after having taken mifepristone in our health facility. We share our experience by describing the model of care and discussing the lessons learned through its implementation.

**Conclusions:**

The program delivered abortion services successfully and required few resources. This paper also reflects on the importance of facilitating SMA in humanitarian contexts. It increases access to care by providing increased confidentiality, close support, ample information, autonomy, and flexibility. It is simple to implement, effective, often preferred by women, and can be linked to information about contraception. The implementation of self-managed models should be expanded, notably in projects that do not have a sexual and reproductive health focus and in restrictive and challenging contexts. It represents a true revolution for access to safe abortion care.

## Introduction

Humanitarian actors increasingly understand the demand for safe abortion care (SAC) and the feasibility of offering it in their interventions [1, 2]. However, access to this care should be drastically improved in fragile and humanitarian settings, and the implementation of interventions in that regard is currently limited.

In order to reduce maternal death and suffering from unwanted pregnancy and unsafe abortion, Doctors Without Borders/Médecins Sans Frontières (MSF) committed to providing safe abortion care and ensuring access to services even in the most remote and challenging areas where we work [3]. In 2022, we provided 44,655 safe abortions in a variety of humanitarian contexts. We have found medically managed abortion to be a game-changer. The simple regimen of mifepristone and misoprostol pills (or misoprostol alone if the former is unavailable) is remarkably easy to use. Decades of research and experience show how safe [4] and effective [5] they are. They successfully end pregnancy more than 95 percent of the time [5] and the risk of severe, life-threatening complication is less than 1 percent [6].

Building on the advantages of abortion pills is self-managed abortion (SMA), which holds the potential of revolutionizing access to care. MSF piloted an SMA model of care based on telehealth in the Middle East. In this piece, we share our experience of providing SMA in a humanitarian setting by describing the model of care, discussing the lessons learned through its implementation, and reflecting on the advantages and challenges of this innovative way to improve access to safe abortion care.

## Safe abortion as self-care

Self-care increases access to healthcare in remote locations, unstable contexts or in areas with poor health infrastructures by limiting the reliance on classic health systems. It represents an opportunity to improve sexual and reproductive health (SRH), as shown by the World Health Organization (WHO), whose first ever self-care intervention guideline in 2019 was developed specifically for SRH [7]. During the COVID-19 pandemic, MSF also called for the increased use of self-care for contraception and SAC [8].

Self-managed abortion is a form a self-care where a woman performs her own abortion outside the medical setting or without clinical supervision. She is a at the centre and a partner in the entire process; she is directly involved in the treatment and chooses to entrust a counsellor to support part of it. An increasing body of literature shows that SMA is safe [9–11] effective [12], acceptable to patients [13] and improves access to care [14].

Health care providers and organizations can assist self-managed abortion in several ways. Community-based provision empowers trained community health workers, traditional birth attendants or similar profiles to provide basic consultations and distribute medication themselves; different initiatives function with accompaniment groups comprised of volunteers [15–17]. Pharmacies that sell abortion drugs can also take on this role [18]. Furthermore, abortion can be supported through telehealth, a form of health service delivery where providers and patients are not interacting in person [19]. For the first time, in its comprehensive abortion guidelines published in 2022, the WHO recommends the use of telemedicine for medical abortion [19]. A relatively common model is the telephone hotline [20]; video calls, online forms [21] and mobile applications are also emerging [22, 23].

## The MSF pilot

### Going beyond the current model of care

Safe abortion care is currently mostly provided in MSF projects that have a sexual and reproductive health (SRH) component. Please confirm the section headings are correctly identified. Typically, women requesting to undergo an abortion are directed from the triage room to meet a designated midwife or gynaecologist, who takes their medical history, defines their gestational age, and performs a consultation. The first pill (mifepristone 200 mg) is taken in the consultation office. To take the second part of the regimen (one or multiple doses of misoprostol, depending on the gestational age), the women are often free to choose if they prefer to be at home or to come back to the facility within 24/48 h.

This configuration limits access to care in several ways: it relies on the presence of specialized staff that are not in every project; it forces the patient to come back in case the appointed person is not on shift; and it instructs the medical consultation to include a physical examination, although it is not necessary [14, 24].

To overcome these barriers, MSF started experimenting with new models of care adaptable to the different low-resource, fragile and/or restrictive contexts we work in. One of those models, tested between January and February 2021 in the Middle East, provided women with abortion medications for self-administration and a telephone number for remote counselling.

### In the Middle East, high needs and low access

In the Middle East, SAC is legally restricted to various degrees across all countries. In some countries, misoprostol is easily available in pharmacies, but it is costly and therefore not accessible to most MSF patients.

MSF included SAC services in several of its projects in the region; however, implementation proved to be challenging for several internal and external factors. When successful, it often necessitated a lot of time and resources (close guidance for the team and specific hard-to-find HR profiles) to set up services and it was easily dropped or interrupted when the team faced hurdles. The pilot was implemented in the region in the hopes of improving our service provision.

The project that was chosen to provide SMA presented cultural, religious and legal constraints to abortion and a high need for access to safe abortion care. In the country of reference, termination of pregnancy on request is legally restricted; therapeutic abortion is allowed in case of foetal impairment and to save the mother’s life. Even for the cases legally allowed, it is difficult to access proper care. Some of the women received by MSF, although they matched the legal criteria to receive SAC, were refused care by private clinics and governmental hospitals out of fear to be labelled as “abortion givers”. As they face such barriers, many turns to unsafe methods. According to an external study conducted in the Middle East in 2018, the women who had induced an abortion mostly relied on undertaking heavy physical activities (60.2%) and using herbal remedies (22.2%) [25], which presents health risks. Furthermore, it was estimated that 48.5% of abortions in the “Western Asia” region, which includes most countries generally understood to comprise the Middle East, are unsafe – 36.3% are “less safe”, meaning that they are either conducted by untrained people or using non-recommended methods, and 12.3% are “least safe”, performed by untrained people using dangerous methods [26].

### Designing the pilot

The MSF in-country team collaborated with a person in charge of sexual and reproductive health technical support, an Arabic-speaking international staff with a midwife background. She managed the program and worked with the identified project team to develop adapted patient pathway and protocols. She also acted as the counsellor who was in contact with the women.

For this purpose, a separate telephone number was activated, free of charge for patients, including international calls. Envelopes were prepared in advance with the phone number, pills, and instructions on how to take them with simple illustrations for people with lower literacy. Two types of envelopes were available: one containing the drugs needed for pregnancies under 13 weeks, and the other containing drugs for pregnancies of 13 weeks and more. They also contained pain management pills.

A specific person was chosen and trained to receive women and provide the brief in-facility part of the care. The staff in the triage (health promoters, translators, nurses and midwives), the midwife supervisor and the team leaders were oriented about the new pathway and how to direct the women to the trained focal person.

### The model of care

Under the pilot, the woman reaches the facility and communicates her need to the triage team, either directly or by asking for “the foreign midwife”, who was in charge of the consultation in the regular model of care. She is then directed to the focal person who simply identifies the gestational age by asking about the last menstrual period – no physical examination nor ultrasound scan is conducted, and no medical history is taken. She gives her the pre-prepared envelope based on the assessed gestational age. The staff member explains that she should call the free phone number in the envelope and records the patient’s number for follow-up by the remote counsellor.

We did not face cases of women who could not access a phone; in case they did not own one, they provided another number (either for their husband’s or a close relative’s) and the counsellor planned a call with her. Over time, some women started to receive the counsellor’s phone number through peers who received the service themselves. In this case, they contacted her directly and only needed to come to the facility to pick up their envelope.

Once the woman is at home, she decides when to call the counsellor and start the process. If she does not call, the counsellor contacts the woman directly though the recorded number. Over the phone, the counsellor proceeds with the consultation (history of the patient), establishes the treatment plan with her (availability of a private space to start the process, how to take the pills, who can support her, how to manage the disposal of the foetus, tissue and placenta) and how to manage the pain. The patient is also asked if she needs additional support (financial, mental health or shelter). The counsellor accompanies the whole SAC process from the first consultation to the last follow-up call (after the abortion is done). She can conduct daily checks if it is desired by the patient, and she remains reachable in case of need.

The medical regimen was orally taken mifepristone (200 mg), followed 24 to 48 h later by misoprostol (800 μg for pregnancies below 13 weeks; for later pregnancies, repeated 400 μg doses every 3 h depending on gestational age, as recommended by WHO guidelines [24]). For first trimester abortions, though 2 doses of 400 μg are required, an additional dose was put in the envelope to be taken under the counsellor’s recommendation in case of incomplete abortion. If they reacted quickly to the first doses, we simply instructed them to get rid of the third one.

### Implementation results

#### Care successfulness

During the 2-month pilot, 22 women received safe abortion care through the self-managed model. As per the MARE Guidelines, which categorize success for medical abortions [27], 19 abortions were successful as the pregnancy was expulsed without need for surgical intervention. Amongst the 3 other procedures, 2 were incomplete and the retained products that were treated with manual vacuum aspiration (MVA), and 1 pregnancy continued and was treated with dilatation and curettage. All patients were able to get a complete abortion.

#### Amount and type of resources needed

This model of care required few resources. The initial preparation was the costliest, as it took time for the counsellor and key project staff to create the new pathway as well as to train the designated in-facility focal person and the staff that would direct patients to her. In terms of human resources, only the counsellor was added to the project set-up. Overall, the pilot reduced the staff’s workload, since most of the tasks previously performed in the health facility (taking the medical history, performing a consultation, discussing treatment, translating between the patient and the international staff member, overseeing the intake of the first and sometimes subsequent pill(s)) were transferred to the counsellor over the phone. Furthermore, the envelopes could be prepared in advance. Regarding expenses, the care also required drugs (in our case, mifepristone and misoprostol) and a SIM card for the counsellor.

### Barriers, enablers, and resulting lessons learned

#### Barriers: flaws to learn from

*Provide the counsellor with a post-paid international SIM card that is free of charge for the patients callin*g. At first, the counsellor’s SIM card did not work for international calls. Sometimes, this prevented patients from reaching her, since she was based in the region but moved in and out of the country of the pilot.

*Put in place a verification process for the envelopes.* By mistake, some did not contain the right amount of misoprostol or pain management pills. The women concerned were able to come back to get more doses, but this could have been more difficult in other contexts.

*Choose a counsellor who understands and can speak the local dialect(s).* Although the counsellor spoke the same language as the patients, their accents and dialects differed, which disturbed the communication.

*Take care of the counsellor’s mental health.* The counsellor’s role is not only to bring medical expertise; she provides emotional support to the patients and she needs to continuously assess a sensitive and risky situation. Those tasks can become heavy and call for proper mental health support.

#### Enablers: strong features to emulate

*Assign a technical support for the counsellor.* à person’s tasks is to follow up on complicated cases, strengthen the remote consultation by providing situational analysis for each context in collaboration with the mission, and ensure monitoring and evaluation of the process to improve quality of care. This also fosters a learning-by-doing approach: as they advise the counsellor, this support person learns, becomes better at facing different cases, and can transfer this knowledge to other projects.

*Ensure the availability of the drugs*. In the country of the pilot, MSF can import both misoprostol and mifepristone through its international supply chain. However, in other contexts, importation is limited, local supply is impossible, or the quality of local drugs is insufficient. A thorough analysis of supply options is essential to avoid service rupture.

*Create an emergency plan for referral* [28]*.* It can be difficult for women to reach a healthcare facility or to go alone. In the initial phone consultation, it is important to establish an emergency procedure as part of the treatment plan. Our patients were asked if they had an available mode of transportation and people to accompany them to the nearest hospital or clinic in case of complications or unease with bleeding and pain.

## Implementing self-managed abortion in humanitarian contexts

### Advantages and challenges

#### A promising way to increase access to safe abortion care

Compared to a configuration of medical abortion where the health provider provides a consultation, counselling, and pill intake supervision in person, SMA has the potential to increase access to healthcare. These advantages are organized below according to the five dimensions of access to care (acceptability, accommodation, affordability, accessibility, and availability) [29].

##### Acceptability

According to our pilot implementation team, feedback from patients show that SMA can be an acceptable way of providing abortion in humanitarian settings. It is a method they are interested in and satisfied with. Some of the main reasons they mention are confidentiality and close support.

SMA provides increased confidentiality, which is crucial in a context where safe abortion care is limited by cultural, religious and/or legal constraints. The fact that patients spend minimal time and number of visits at a health facility reduces the risk – both real and perceived – of their abortion being discovered or judged by other patients, staff, or relatives. The ability of SMA to provide privacy can have an enormous impact on women accessing care. Beyond MSF projects, this has been observed by organizations such as Marie Stopes International (MSI): feelings of embarrassment and fears of stigmatization when seeking care can lead women to choose unsafe abortion methods over safe care providers [30]. Many of their patients would not have been comfortable attending consultations in person and might have chosen methods that would jeopardize their health if it was not for telemedicine [31].

SMA also has the advantage of facilitating access to information and support. In India, the Ipas Development Foundation found that the main need for women who self-managed their abortion was accurate information [32]. When the process is supported, women are in direct contact with the healthcare provider; therefore, they can reach them easily to ask questions or be reassured about the abortion process. In testimonies of patients who used SMA, close support by the counsellor plays a prominent role in their satisfaction [31]. For instance, a woman from the Middle East pilot told us: “the MSF doctor supported me the whole time. I would call her, and she would answer [the phone], even in the middle of the night. From 4:00 a.m. to when the abortion was completed at 3:00 p.m., she was frequently checking up on me. I felt very good when I was able to end the pregnancy safely.” [33] It has been demonstrated elsewhere that SMA enables a supportive experience for women. For instance, in the legally restrictive context of Indonesia, a study on SMA provided with a hotline found that patients’ “levels of preparedness, confidence, and feelings of support were all extremely high” [34].

##### Accommodation

Furthermore, self-managed abortion allows patients to make the decision of the timing and location of the care. This accommodation to their lives is important for confidentiality reasons, but also for personal logistics. Women might have difficulties to stay away from home for a long period of time or to leave repeatedly due to their economic and familial responsibilities; furthermore, they most often already have children to take care of [33].

##### Affordability and accessibility

Remote support also makes care more affordable and accessible. It reduces its indirect costs, as can be expensive to travel multiple times to a health facility to take pills, have a follow-up consultation, or come back to start the process because no qualified practitioner was present at the first visit. Furthermore, it facilitates access to care for people living far away from a facility that provides abortions, which is the case in low-resource settings.

##### Availability

A self-managed approach increases availability of the service, which is especially important in a context where its provision is restricted. As one of the patients in this pilot told us: “I finally went to the MSF hospital. It took me one month to find a facility that would help me.”[33] In the model of care used by MSF, anyone with adequate training can give women the envelope, since the part of the care that requires specialized expertise is done remotely. Consequently, it is simple to train more than one project staff and to ensure full-time ability to provide the envelope. To facilitate this training, MSF partnered with the organization HowToUseAbortionPill.org to create short online courses for humanitarian aid workers [35]. Material was also created for women who manage their abortion [36].

#### An easy model to implement

SMA is well suited to the context of humanitarian aid. Indeed, as shown by our pilot, it requires few resources. By any means, medical abortion is cheaper than surgical abortion, notably because misoprostol is very affordable, and it is the only indispensable drug for this process. Moreover, if medical abortion is already provided in a project, switching to a remote model reduces the workload of specialized staff and the dependency on their physical presence. Another advantage is that it can be put in place in projects that do not provide SAC, and in projects that do not have a sexual and reproductive health component. SMA can be implemented anytime – at the beginning, during, or at the end of a project – and in a variety of settings – such as low-resource, legally or culturally restrictive, and/or emergency. It thus represents a potential breakthrough for safe abortion care provision.

#### A program that still allows to discuss contraception

In the area of the pilot project, women regularly use induced abortion as a contraceptive method. Several studies conducted in the region between 2013 and 2020 show that the attitude and the satisfaction of the women towards contraception is linked to the level of information they received from healthcare providers. It is thus essential to integrate thorough and informative discussions about contraception within abortion care. Self-managed models allow it just as well as more traditional configurations. In all projects where MSF has implemented forms of SMA, we were able to provide this information to women who received care. We also provided contraceptives if they were interested, though it was not conditional to care, and their uptake was high.

#### An effective method, yet not perfectly

Research has shown that self-managed abortion is effective. In England, a study compared a cohort that received a traditional medical abortion (in-person consultation, ultrasound scan, and administration of mifepristone, and misoprostol to be taken at home) and a “telemedicine-hybrid” cohort who had a phone or video consultation and took the entire regimen at home. Though it is important to note that only women under 10 weeks of pregnancy could be part of the latter group, which is associated with less risks of complications [37], the results remain impressive: the rates of successful medical abortion were 98.2% in the traditional cohort and 98.8% in the remote group [14]. A study conducted in the legally restrictive contexts of Nigeria and Argentina showed that self-managed abortion was as effective as medical abortion done in a clinical setting [38].

Though we still lack research in humanitarian settings, MSF explored various models of SMA and the outcomes have been successful. In this Middle East pilot, 86% of abortions were successful without surgical intervention. In another MSF project, which took place with sex workers in Africa for three years, community based SMA was provided to 423 women and this success rate was 97%.

However, as attested by research and confirmed by MSF’s experience, SMA is not 100% effective. In this pilot, one person did not respond to the drugs even by taking all the indicated misoprostol doses and needed to come back to renew the dosage. Also, surgical procedures were needed in some cases; therefore, the project needed to have these resources internally or to be able to refer patients. This illustrates that, though SMA is suited to non-SRH projects or in settings with low medical resources, it is not a panacea and should be implemented with these constraints in mind.

#### A configuration that women often prefer, yet not always

According to MSF’s experience, most women prefer self-managed over in-facility abortion. However, we also noted that it is not some patients’ preference for various reasons – fear of pain, stress because it is their first pregnancy, desire for a rapid procedure, etc. In Cambodia, Marie Stopes International reports that many women prefer surgical abortion because it is faster, causes less bleeding, and does not require them to carry materials that could be discovered and impair their privacy [30]. This has been seen in MSF projects as well. SMA is therefore not meant to completely replace other methods but should be seen as a promising tool to add to health providers’ arsenals.

### Avenues for future research and practice

To continue progress on the provision of self-managed abortion in humanitarian settings, more pilots need to be put in place, including at a larger scale and, as is increasingly the case in MSF, in projects without a sexual and reproductive health component.

Other SMA models should be tested, such as fully remote configurations or community-based care. For instance, the model presented here could be adapted by exclusively providing the phone number through word-of-mouth and local partners, and by delivering the drugs to women outside the clinic. Also, in other projects, MSF has worked with community health workers and peer educators to ensure that people living in remote areas could access SAC without having to travel to a health facility.

Attention should also be given to partnerships with local organizations, and different modalities of task-sharing and transition should be explored. For instance, the same model was implemented in other projects in the Middle East, but a different version in which a local organization conducts the remote consultation was put in place. If the partner’s resources allow it, this type of collaboration can be a way to ensure continuity of care if the counsellor is not available, or to replace her altogether. This approach is also important for sustainability, as international humanitarian organizations will eventually leave the project location.

As noted by other actors, many considerations determine the relevance of SMA in humanitarian contexts. Some of them, like access to water and sanitation and adequate housing, were not mentioned in this paper [2]. It would be valuable for other experiences to address these constraints and to share lessons learned to improve practice.

## Conclusion

There is not only one answer on the provision of safe abortion care, but multiple possibilities to be explored. One of the ways humanitarian organizations and health providers in humanitarian and fragile settings can improve safe abortion care is by supporting self-managed configurations. The pilot conducted by Médecins Sans Frontières in the Middle East, which provided drugs for self-administration and telehealth counselling via a telephone hotline, shows that this model of care can be replicated: it can be delivered successfully and requires few resources. Self-managed abortion increases access to care by providing increased confidentiality, close support, ample information, autonomy, and flexibility. It is simple to implement, effective, often preferred by women, and can be linked to information about contraception. The implementation of self-managed models should be expanded, notably in projects that do not have a sexual and reproductive health focus and in restrictive and challenging contexts. It represents a true revolution for access to safe abortion care.

## Data Availability

Since the pilot was not originally built as a study, some data cannot be shared publicly. Because of ethical constraints and confidentiality about the project in question, we cannot share data about patient feedback beyond publicly available testimonies, nor references to the exact context. All shareable, non-personal data from the described pilot are included in this published article. No datasets were generated or analysed during the current study.
